# Professor W. B. Miller

**Published:** 1890-11

**Authors:** 


### Editorial.

### PROFESSOR W. B. MILLER.

Unusual space has been given this month to the reports of banquets recently held in different sections. The remarkable ovations that have met Professor Willoughby B. Miller, since his advent to our shores, have a significance that is at once an honor to him as they are interesting as a mark of progress in the manner of treating our original workers and thinkers. It has been given to but few scientific men to receive such marks of approbation. The walks of science are in the silent cloisters,—in the laboratories,—away from the plaudits of men; indeed, it may be supposed the very entrance of possible praise or blame of an outside world would be inimical to true investigation. The seeker after truth needs no incentive but the value of results. The ambition of the scientist lives on what is developed, and even the thought of the intruder is a disturbing element.

Hence, it is very true, that the spontaneous ovation of esteem must have been in the light of a surprise, and, perhaps, in some measure not quite comprehended, as the honest investigator is certain always to undervalue the results attained, and looks upon them merely as stepping-stones to something higher.

While these remarks are unquestionably true, the present outburst of feeling is at once gratifying, and in a measure represents a growing conviction that the time has come to recognize the recluse of the laboratory as the man of the hour, the one who is erecting the foundations for all the knowledge we possess, or are likely to come into possession of,—the "house we live in." It is, therefore, significant as a time of mental growth, and celebrates this quite as much as it means honor to the man.

This is the view in the broader sense of scientific work; but in the more limited range of dental labor it means even more than that, as it is the marking of an epoch, the celebration of a period when the dreams of a past century have reached an actual realization. It is the culmination of the work of many. It means, if it means anything, that a long and difficult problem has been solved.

It means that the opprobrium of the dental and medical profession (for they are about equally involved) has been removed, and that by a dentist. That this is worthy of rejoicing along with the other great events of history must be clear to the average mind.

The fact brings up, however, this important reflection, a point that never should be lost sight of,—that no one man does the whole work in a given line of investigation. This can be said without disparaging in the least the results attained by any. The work of the world is an aggregation. It is developed from the simple atoms of thought to the ultimate perfected truth.

When the scientific mind of the dental profession examines the steps that have led to this elucidation of dental caries, the impression must be strongly felt that it has been a progressive development from the time that Leeuwenhoek (1722) demonstrated, with very imperfect means, the existence of low forms of life. From which gradually grew the idea that the invisible life was a powerful factor in changing conditions in the visible world, a fact freighted with immense results for the future life of peoples, and which led up to the remarkable work of the present.

In the special subject of dental caries we trace a direct series from Ficinus (1846), with his "Denticolæ," which became "Bühlmann fibres" by union, to Tomes (1848), who found a "peculiar vegetable growth that infested carious teeth," to Klenke (1850), with his purely inflammatory dental caries, "protococcus dentalis," and putrefaction, and, last, chemical action. From Klenke to Leber and Rottenstein, with their brilliant monograph, that stirred the dental world as nothing else previous to the Miller papers. Then came the valuable work of Milles and Underwood, and from this to the subject of this article. Each of those mentioned performed a portion of the labor that led to the final conclusions. It is, therefore, proper that we should recall the underlying work of these men, some pioneers, and others contemporary with Dr. Miller. In a review of the past and present, let us not forget also the labors of others not already mentioned,—of Ehrenberg, Neumann, Wedl, Weil, Walkoff, and Koch, in Germany, Pasteur and Magitot in France, and of Clark, Black, and Abbott in the United States.

The elucidation of the mysteries of life is but in its infancy. The problems may never be wholly solved; but when one step has been finished it is our duty to stand upon it, and, looking forward, give honor to the builder.

